# Carbon threads sweat-based supercapacitors for electronic textiles

**DOI:** 10.1038/s41598-020-64649-2

**Published:** 2020-05-07

**Authors:** Nuno Lima, Ana C. Baptista, Bruno M. Morais Faustino, Sofia Taborda, Ana Marques, Isabel Ferreira

**Affiliations:** CENIMAT/i3N, Departamento de Ciência dos Materiais, Faculdade de Ciências e Tecnologia, Universidade Nova de Lisboa, Caparica, 2829-516 Portugal

**Keywords:** Energy science and technology, Energy storage

## Abstract

Flexible and stretchable energy-storage batteries and supercapacitors suitable for wearable electronics are at the forefront of the emerging field of intelligent textiles. In this context, the work here presented reports on the development of a symmetrical wire-based supercapacitor able to use the wearer’s sweat as the electrolyte. The inner and outer electrodes consists of a carbon-based thread functionalized with a conductive polymer (polypyrrole) which improves the electrochemical performances of the supercapacitor. The inner electrode is coated with electrospun cellulose acetate fibres, as the separator, and the outer electrode is twisted around it. The electrochemical performances of carbon-based supercapacitors were analyzed using a simulated sweat solution and displayed a specific capacitance of 2.3 F.g^−1^, an energy of 386.5 mWh.kg^−1^ and a power density of 46.4 kW.kg^−1^. Moreover, cycle stability and bendability studies were performed. Such energy conversion device has exhibited a stable electrochemical performance under mechanical deformation, over than 1000 cycles, which make it attractive for wearable electronics. Finally, four devices were tested by combining two supercapacitors in series with two in parallel demonstrating the ability to power a LED.

## Introduction

Wearable electronic devices have emerged in the last decade due to a remarkable interest in intelligent textiles or electronic skin applications. Transistors, memory devices, organic light emitting diodes, triboelectric and energy storages devices have been produced in flexible substrates enabling conformity to curved surfaces^1^. Among the storage devices proposed, the fibre device is the most attractive as it can be incorporated directly on textiles or other wearable applications^2^. Therefore, numerous works have focused on the development of fibre-based capacitors and supercapacitors^3–6^. These devices can be produced in sheets and rolled out to have a fibre geometry. Kim and co-workers^7^ have tested a carbon fibre paper as electrode reaching a specific capacitance of 140 F.g^−1^ and 90% of capacitance retention after 5000 cycles using 0.5 M H_2_SO_4_ as electrolyte and Ag/AgCl as the reference electrode. They have further improved these performances with decorated Polypyrrole-NiCo_2_O_4_ carbon paper^8^ reporting specific capacitance of 900 F.g^−1^ (at 1A), and 88% of capacity retention (5 A.g^−1^) after 10,000 cycles. Silver nanodendritic cellulose acetate electrodes were tested by Kesavan Devarayan^9^ in the form of flexible sheets showing specific capacitance in the range of 125 F.g^−1^ after 1000 cycles and a capacitance retention around 20%. Single-walled carbon nanotube/polyaniline (SWCNT/PANI) nanoribbon paper with volumetric capacitance (40.5 F.cm^−3^) and mechanical compliance toward bending and folding, maintaining electrochemical stability up to 1000 cycles has been reported by Dengteng Ge^10^. The fabrication of a yarn of carbon nanotubes filaments with 23 µm diameter for electrodes of supercapacitors has been reported by F. Liu^11^ and co-workers.

Pristine twisted carbon fibres (CFs) coated with a thin nickel-cobalt double hydroxide (Ni-Co DHs) layers were applied to flexible fibre-shaped supercapacitor obtaining specific capacitance up to 1.39 F.cm^−2^ in KOH aqueous electrolyte^12^. Chenyang Yu^13^ and colleagues have developed an electrochemical method to directly modify CFs into high performance electrodes. Two parallel modified CFs electrodes delivered an improved capacitance when compared to pristine CFs (87.2 F.cm^−3^ at 1.0A) using PVA.H_2_SO_4_ as electrolyte. Cellulose fibres with open pore channels extended along the fibre with a specific capacitance of 250 F.g^−1^ in Na_2_SO_4_ electrolyte retaining 85% of its capacitance up to 50,000 cycles have been published by Gui *et al*.^14^.

Energy-storing devices, such as fabric supercapacitors, are ideal for wearable electronic applications, since it is lightweight, can be cheaper and mechanically compatible with textiles. However, electrolytes such as KOH and PVA.H_2_SO_4_ are incompatible with most of the wearable purposes. Energy harvesting devices working with sweat solution as the electrolyte to generate electricity are the most compatible route to achieve a body wearable source of energy. A highly flexible and symmetrical wire-based supercapacitor has been studied using a carbon-based wire as inner electrode, cellulose acetate electrospun fibres as the separator, and outer electrode carbon-based wire twisted around the separator. The carbon wire surface was enhanced by *in-situ* polymerization of pyrrole (Py) to improve the electrochemical performances of the supercapacitor. Electrochemical performances of carbon-based wires with and without polypyrrole (Ppy) functionalization were analysed using a simulated sweat solution as electrolyte.

This work reports for the first time on the development of a highly flexible fibre-shaped supercapacitor able to use the wearer’s sweat as the electrolyte. The use of electrolytes based on bodily fluids shows excellent potential for electronic textile applications.

## Experimental section

### Preparation of carbon-based wires as electrodes

Commercially available carbon thread (from TENAX, 218 Ω/m) was used as received. Further functionalization to improve its performance was achieved by coating the fibres with polypyrrole (Ppy). *In situ* polymerization of pyrrole (C_4_H_5_N, Sigma-Aldrich, Mw = 67.09 g.mol^−1^, purity = 98%) on the surface of carbon wire was carried out using FeCl_3_.6H_2_O (Sigma Aldrich) as the oxidizing agent according to the protocol reported on previous work^15,16^. The carbon wires (~ 4 cm long and 180 µm thick) were immersed in an aqueous solution of pyrrole, 0.05 M for 10 min under magnetic stirring. Then, an aqueous solution of FeCl_3_.6H_2_O was gently added to monomer solution considering a monomer to oxidizing agent mass ratio of 2 and a reaction time of 20 h. After that time, the carbon-based wires were thoroughly washed with distilled water and ethanol in order to extract the by-products and residues of the reaction and left to dry under ambient conditions.

### Preparation of electrospun fibres as the separator layer

A 12% wt cellulose acetate (CA) solution was made by dissolving CA (Sigma-Aldrich, Mn = 61,000 with 40% acetyl groups) in acetone and dimethylacetamide (DMAc) in a 2:1 (wt) proportion. The solution was loaded onto a 1 mL syringe (B. BRAUN) with a 21-gauge needle tip (from ITEC). Previously reported parameters^15^ were used to produce the electrospun membrane. The process was carried out under controlled environmental conditions, namely at temperature of 22 °C and relative humidity of 40%, approximately. A thermoplastic frame, with 6 cm ×6 cm of dimension, was designed using Autodesk Fusion 360 software and printed in a 3D printer (PRUSA i3) to support the carbon-based wires (inner electrodes) during the electrospinning process (Fig. SI1). For 60 minutes deposition, a CA fibres layer with a thickness of 193 ± 74.9 µm, approximately, was obtained.

### Assembly of the supercapacitors with twisted configuration

A twisted configuration was selected for wire supercapacitor assembling. A detailed schematic is showed in supporting information, Fig. SI2. After the deposition of CA electrospun fibres on carbon or carbon functionalized wire, other carbon-based wire was manually coiled around the separator. Several combinations of inner/outer electrodes were studied: carbon thread/carbon thread (denominated by CT/CT); carbon thread functionalized with Ppy/carbon thread functionalized with Ppy (denominated by CTf/CTf), and an asymmetrical configuration of carbon thread functionalized with Ppy/carbon thread (denominated by CTf/CT). The influence of the number of twists on specific capacitance obtained for wire supercapacitor was also evaluated.

### Electrolyte preparation

Two electrolytes were studied to evaluate the electrochemical performances of supercapacitors here proposed – KOH and a simulated sweat solution. An aqueous solution of 0.1 M KOH from EKA was prepared with ultrapure water (pH** =** 13). Additionally, a simulated sweat solution (SSS) was prepared according to the procedure referred in literature^17^ (according to ISO105-E04:2013). 0.05 g of L-histidine (Sigma Aldrich, 99%), 0.5 g of sodium chloride (Sigma Aldrich, 99.5%), and 0.22 g of sodium phosphate monobasic (Fluka analytical, 90%) were dissolved in 100 mL of ultrapure water (pH** =** 5.5). After prepared, the solution was kept on the fridge.

### Electrochemical characterization

The electrochemical characterization was carried out by cyclic voltammetry (CV), galvanostatic charge and discharge cycles (CD) and electrochemical impedance spectroscopy (EIS) using a potentiostat Gamry Instruments-Reference 3000. All experiments used a two-electrode setup (Fig. SI3).

The influence of electrolyte solution (KOH and SSS) and twisting number on the specific capacitance of CT/CT, CTf/CT and CTf/CTf was evaluated by cyclic voltammetry at a scan rate of 100 mV.s^−1^. CT/CT, CTf/CT and CTf/CTf supercapacitors (with a twisting number of 3) were submitted to 10 cycles from −0.5 V to 0.5 V at different scan rates (20, 40, 60, 80, 100, 200, 500 and 1000 mV.s^−1^) in using 40 µL of SSS. The specific capacitance was determined using the 8th cycle for each scan rate following Eq. 1:

1 $$\frac{C}{m}=\frac{1}{m\ast v\ast \Delta V}\ast \int {I}_{(V)}dV$$

where $\frac{{\boldsymbol{C}}}{{\boldsymbol{m}}}$ (F.g^−1^) is the specific capacitance, ***m*** (g) is the electrode’s mass, ***v*** (mV.s^−1^) is the potential scan rate, $\Delta {\boldsymbol{V}}$ (V) is the potential sweep window and ***I***(_*V*_) (A) is the resulting current. Results were extrapolated from a specific potential window.

The CTf/CTf device was submitted to different bending angles (30°, 60°, 90° and 180°) to ensure that the device maintains its performance under strain. Cyclic voltammetry curves were measured as a function of the bending angle, which was varied by curving the supercapacitor wire towards the different angles. The specific capacitances were determined from the 8th cycle. The stability of the electrochemical performance of the wire over consecutive sets of bending cycles was also accessed. The bending angle of the supercapacitor wire was repeatedly varied from 0° to 180° in a constant pre-defined step using a computer controlled home-made apparatus. One cycle is defined for each 180° angle variation, alternatively measured in clockwise and anticlockwise directions. Cyclic voltammetry curves were measured every 100 cycles to determine the specific capacitance of the device as a function of the number of cycles (1000 cycles).

The electrochemical impedance spectroscopy (EIS) experiment was performed with 100 mV rms of AC voltage and with 40 µl of SSS, from 1 MHz to 10 mHz. The ionic conductivity of the electrolyte, KOH and SSS, in the CA electrospun membrane for different frequencies was determined by EIS using Eq. 2 (Fig. SI5). A cellulose acetate electrospun membrane, with a thickness of 100** ±** 10 µm, was impregnated with 10 μl of the electrolytic solution and sandwiched between two gold electrodes.

2 $${\boldsymbol{\sigma }}=\frac{{\boldsymbol{l}}}{{\boldsymbol{Re}}({\boldsymbol{Z}})\ast {\boldsymbol{A}}}$$

where σ is the ionic conductivity, l is the distance between electrodes, Re(Z) is the measured impedance and A is the electrodes contact area.

Cycling stability was studied by subsequent charging/discharging tests. 1300 charge/discharge cycles were performed to CTf/CTf supercapacitor at a constant current density of 78 mA.g^−1^ (40 seconds each cycle). The device was partially immersed in 5 ml of electrolyte to guarantee that the separator was always soaked with the electrolyte until the end of the test to discard the effect of water evaporation with time on capacitance. An active mass of 6.41 mg was considered for capacitance calculations.

To calculate the specific capacitance (F/g), energy density (E, kW/kg) and power density (P, Wh/kg) from the curves, the following equations were used^18^:

3 $$\frac{{\boldsymbol{C}}}{{\boldsymbol{m}}}=\frac{{\boldsymbol{I}}\ast \Delta {\boldsymbol{t}}}{{\boldsymbol{m}}\ast \Delta {\boldsymbol{V}}}$$

4 $${\boldsymbol{E}}=\frac{{\boldsymbol{C}}\ast {(\Delta {\boldsymbol{V}})}^{2}}{2}$$

5 $${\boldsymbol{P}}=\frac{{\boldsymbol{E}}}{{\boldsymbol{t}}}=\frac{{\boldsymbol{I}}\ast \Delta {\boldsymbol{V}}}{2}$$

where C/m is the specific capacitance, m (g) is the electrode’s mass, ∆V (V) is the sweep potential window, I is the applied current and ∆t (s) is the discharge time.

Several wire-based supercapacitors were integrated in series and in parallel configurations to evaluate their ability to meet different power requirements. Charge/discharge measurements were carried out for each configuration using a constant charge/discharge current of 0.5 mA.

Additionally, to demonstrate their ability to turn on a green light emitting diode (LED) that operates at a minimum voltage of 1.8 V, two devices were connected in series with a crocodile clip. The loose terminal from each device was then connected to two different crocodile cables, which were used to charge the devices with a 3 V lithium coin cell and then to power the green LED. Lately, two more supercapacitors were connected in parallel with the previous ones for the same purpose.

### Morphological and chemical characterization

The morphology of samples was studied by scanning electron microscopy (SEM) (model Hitachi S2400). Samples were placed in the sample holder using a carbon tape and then coated with a thin layer of gold-palladium alloy. Confocal Raman spectrophotometer (Witec Alpha 300 RAS) using a laser of 532 nm and 1.6 mW of power was used to demonstrate the chemical composition of the supercapacitor wire.

## Results and discussion

**Figure 1:**
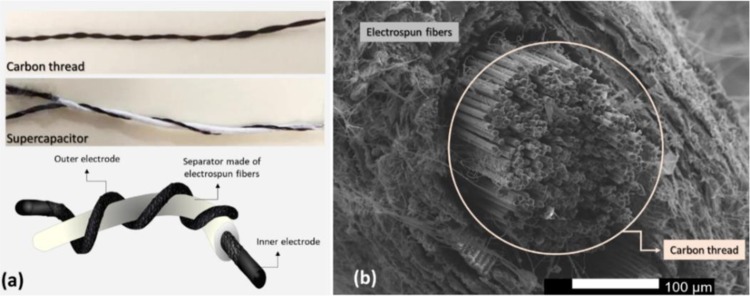
Twisted configuration wire-based supercapacitor: (**a**) Photography and schematic of carbon thread and supercapacitor; and (**b**) SEM image of carbon thread coated with electrospun fibres.

Fibre-like supercapacitors were fabricated with the outer electrode in a twisted configuration as indicated in Fig. 1a. A commercially available carbon thread was used as inner and outer electrodes of the proposed devices. A thin layer (approximately 193 ± 74.9 µm) of cellulose acetate electrospun fibres were deposited on the surface of the inner electrode as the device separator. The SEM image of the commercial carbon thread (inner electrode) coated with CA electrospun fibres is depicted in Fig. 1b. A carbon thread (CT) composed of carbon multi-filaments can be observed in the core of a cellulose-based electrospun fibres matrix. *In situ* polymerization of pyrrole was made onto the surface of the carbon threads (carbon thread functionalized, CTf) in order to improve their electrochemical properties. Symmetrical (CT/CT and CTf/CTf) and asymmetrical (CTf/CT) inner and outer electrodes combination was evaluated.

Generally, KOH and PVA.H_2_SO_4_ electrolytes are the most commonly studied for wearable supercapacitors since they have good ionic conductivity. However, considering possible direct contact with skin, their integration into wearable textiles can be toxic and unsafe. In this work, the possibility of using sweat as an electrolyte was investigated. Body sweat contains various ions, amino acids, proteins and lipids; however, except for ionic species, most of the components present in sweat are neutrally charged and have a negligible effect on ionic conductivity^19^. An artificial sweat solution (SSS) was prepared and the fibres were impregnated with 40 μl from the respective solution. The same procedure was followed using KOH as the electrolyte to compare the performances of different supercapacitors structures. The specific capacities obtained for each supercapacitor and electrolyte are presented in Table SI1.

Considering the same amount of electrolyte swollen by the fibres, we observed that specific capacitance of devices with SSS is comparable to KOH and independent of the structure used. Symmetrical CTf/CTf device displayed the highest values of specific capacitance either for KOH or SSS electrolyte when compared with CT/CT and CTf/CT supercapacitors possibly due to the increase of surface area available for ion adsorption.

Generally, the higher capacitance was observed when SSS is used as electrolyte which can be explained by its higher ionic conductivity within the CA membrane. SSS and KOH solutions notably differ in their pH values (5.5 and 13, respectively). As such, the degree of protonation of the available hydroxyl groups of the CA structure will differ considerably – with a CA structure less protonated being prone to higher values of ionic mobility at lower frequencies (Fig. SI5).

**Figure 2:**
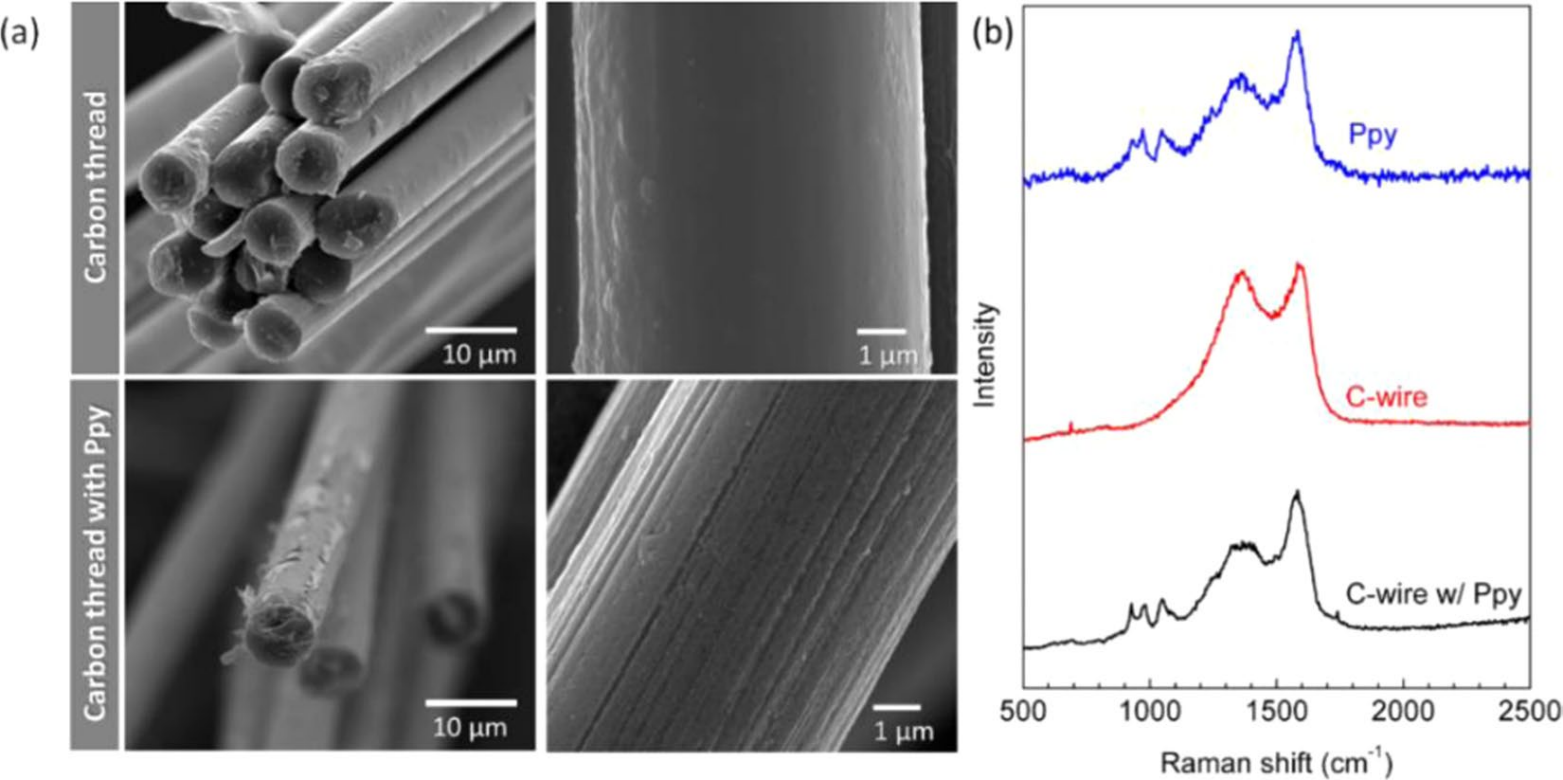
(**a**) SEM images and (**b**) Raman spectra of the carbon threated wire before and after coating with Ppy. Raman spectrum of pure Ppy powder is also represented in blue for comparison.

The surface morphology of carbon thread before and after Ppy functionalization is depicted in Fig. 2a. The carbon thread consists of multi-filaments of carbon with an average diameter of approximately 6 µm each and with a smooth surface. After the *in-situ* polymerization of Py onto the surface of carbon thread, the formation of a Ppy nanocoating (Fig. SI4) becomes evident with the consequent surface roughness increasing. This may explain the higher surface area and the improved electrochemical performances obtained for CTf/CTf structure.

Raman spectra of carbon thread before and after Ppy functionalization are presented in Fig. 2b in red and black, respectively. For comparison, the Raman spectrum of pure Ppy polymer is also included in Fig. 2b in blue.

The Ppy characteristic peaks at 930, 980, 1048, 1365 and 1581 cm^−1^ have been respectively assigned to the bipolaron ring deformation, polaron symmetric C–H in plane bending vibration, ring stretching and C=C stretching vibration modes^20^. The latest two peaks are the most dominant Raman peaks in all the carbon materials spectra, typically assigned to the D and G bands, centred at 1350 and 1597 cm^−1^, respectively. The G band is characteristic of the sp^2^ carbon atoms in the 2D hexagonal lattice and the D band is attributed to structural defects and disorder in the hexagonal lattice. The D and G peaks in the Raman spectra measured from the C thread wire before and after coating with Ppy are centred at about the same wavenumbers, but their intensity ratio (ID/IG) was altered.

**Figure 3:**
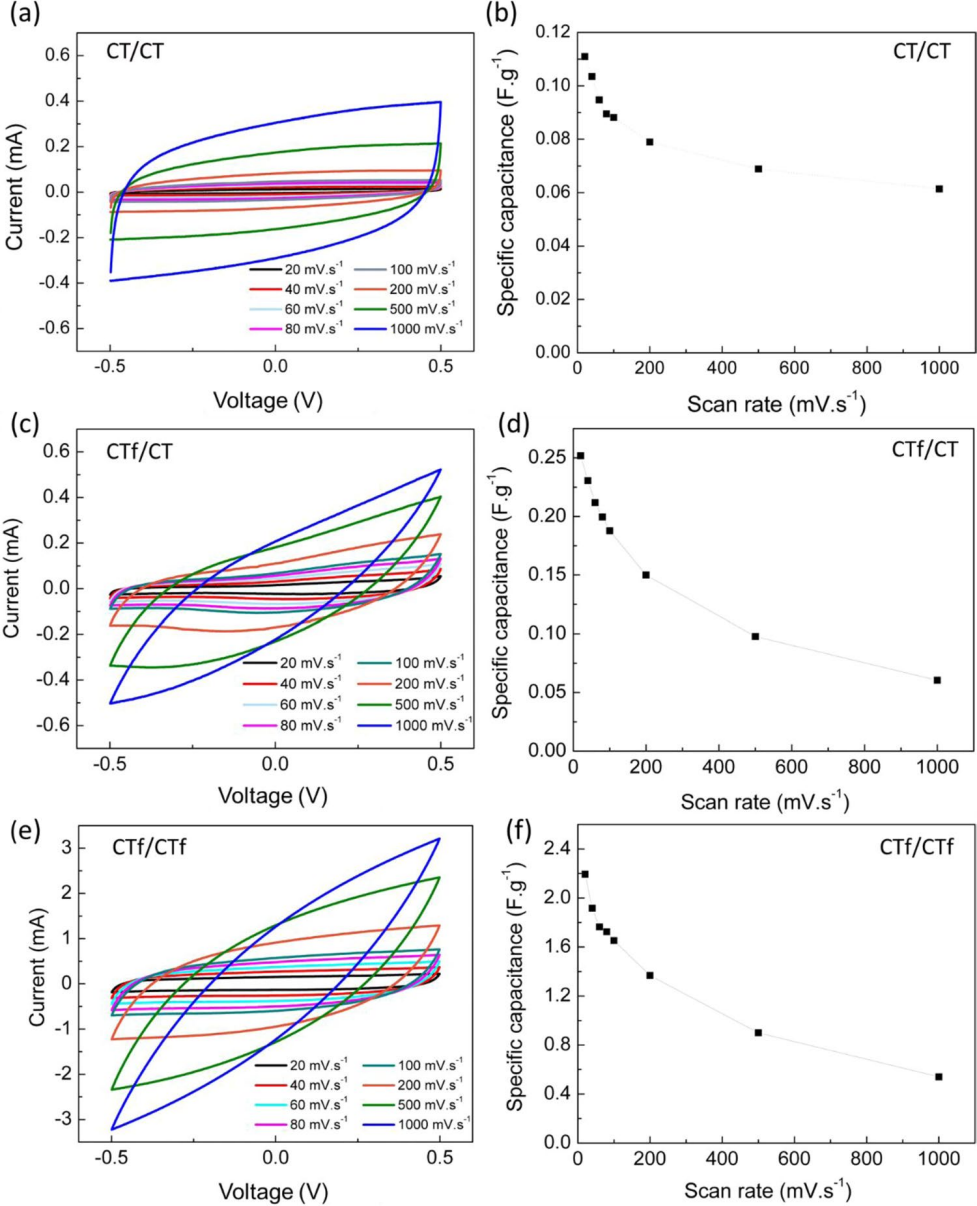
Cyclic voltammetry curves and specific capacitance obtained at different scan rates: (**a**,**b**) for CT/CT; (**c**,**d**) for CTf/CT; (**e**,**f**) for CTf/CTf.

The electrochemical properties of CT/CT, CTf/CT and CTf/CTf supercapacitors were studied by cyclic voltammetry (CV) using a two-electrode configuration and SSS as electrolyte and 10 consecutive cycles of cyclic voltammetry from −0.5 V to 0.5 V using different scan rates (20, 40, 60, 80, 100, 200, 500 and 1000 mV.s^−1^). The specific capacitance was determined using the 8^th^ cycle obtained for each scan rate. Both CV curves and specific capacitance are shown in Fig. 3. The voltammograms obtained for CT/CT supercapacitor exhibit the quasi-rectangular shape typical of a double-layer capacitor at all scan rates showing a high-rate performance (Fig. 3a,b). For instance, at 100 mV.s^−1^ the device displays a specific capacitance of 0.09 F.g^−1^.

By functionalizing the inner electrode with Ppy, the electrochemical performance of the device is improved (Fig. 3c,d). The CV curves demonstrate an asymmetric deformed rectangular shape at scan rates from 20 to 100 mV.s^−1^, exhibiting a specific capacitance of 0.19 F.g^−1^ at 100 mV.s^−1^. However, at higher scan rates, an increase in resistivity is observed which results in a rapid decrease of the capacitive behaviour due to an incomplete electrical double layer formation.

CV curves obtained for CTf/CTf preserved the symmetric reversible quasi-rectangular shape from 20 to 200 mV.s^−1^ demonstrating a good scan rate capability and fast ions transportation (Fig. 3e). Improvement of specific capacitance is observed (Fig. 3f) when compared to previous structures, 1.66 F.g^−1^ at 100 mV.s^−1^. This enhancement can be explained by the small particle size of Ppy (79 ± 16 nm) that enables an enlarged electrolytic surface area with more active sites for ionic species during intercalation or electrosorption, indicating a possible pseudocapacitive contribution of Ppy. It was estimated a loading Ppy mass of 1 mg, approximately, for a wire of 4.5 cm-long, expecting that the performance of CTf/CTf may increase with the loading amount of Ppy content since it enables an enlarged active surface area. A similar resistive behaviour comparable with CTf/CT is also observed at high scan rates.

**Figure 4:**
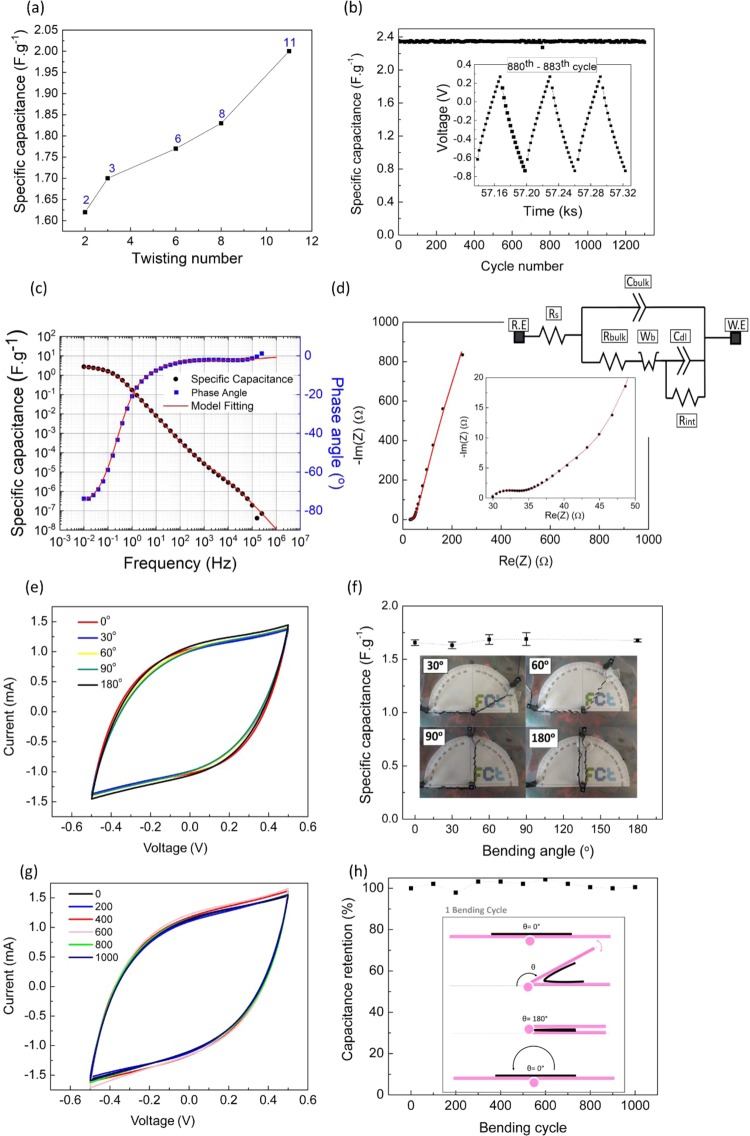
(**a**) The influence of the twisting number on capacitance; (**b**) Specific capacitance obtained for 1300 charge/discharge cycles at a constant current density of 78 mA.g^−1^. The device was immersed in 5 ml of SSS and an active mass of 6.41 mg was considered for capacitance calculations; **(c**) Bode plot and (**d**) Nyquist plot from CTf/CTf at 100 mV rms AC with the specific capacitance; Cyclic voltammetry curves obtained with a scan rate of 100 mV.s^−1^ at different bending angles and after performing sets of bending cycles up to 1000 (**f**). The corresponding specific capacitance as a function of the bending angle is shown in (**g**) and the capacitance retention as a function of the number of bending cycles in shown in (**h**), which illustrates the bending angle and its variation over 1 bending cycle.

The specific capacitance increases with the increasing number of twists of outer electrode around the separator of CTf/CTf device due to the enhancement of electrode surface area and consequent ions adsorption (Fig. 4a). Cycling stability was evaluated by charge/discharge (CD) measurements at 78 mA.g^−1^ for 1300 consecutive cycles (Fig. 4b). Typical CD curves from a supercapacitor were obtained at a constant charge current of 0.5 mA for 30 seconds and a discharge current of −0.5 mA for 30 seconds. The specific capacitance was determined for each cycle showing a capacitance retention of about 100% after 1300 cycles which indicates a good cycling stability behaviour. The CTf/CTf structure displayed a specific capacitance of 2.3 F.g^−1^ for potentials from 0.82 V to 1.10 V, and energy of 386.5 mWh.kg^−1^ and a power density of 46.4 kW.kg^−1^.

Electrochemical impedance spectroscopy (EIS) was performed from 1 MHz to 10 mHz on CTf/CTf device. Figure 4c shows the bode plot with a fitting model adapted from the literature^21^. From the bode plot, a phase shift of −73.81° at the low frequency region is observed, which indicates a capacitive behaviour. At the high frequency region, the device behaves as a resistor due to the lack of interfacial processes^21^. In this region, the electrical current must overcome the device’s electrical resistance, which can be credited to a surface resistance (R_s_) of 29.34 Ω (attributed to the electrodes, electrolyte, separator and external circuit resistances). The ion movement sourced from the bulk electrolyte leads to the formation of a capacitance parallel with a resistance (R_bulk_ and C_bulk_), that is more pronounced between 10 Hz and 1 kHz with a value of 6.88 Ω. The sum of R_s_ with R_bulk_ results in the small semi-circle shown on the Nyquist plot (Fig. 4d), indicating low ion resistance in the bulk of electrolyte (separator). With decreasing frequency, the specific capacitance increases and the phase angle decreases indicating a superior ion movement. The linear region with an approximate 45° slope (from 35 to 45 Ω) is a consequence of the ion diffusion impedance (Warburg impedance, Wb) which models the movement of ions through the separator layer, into the interface.

At 10 mHz the obtained specific capacitance is 2.74 F.g^−1^ which according to the used circuit model is a consequence of the fully charged electrical double layer capacitance (C_dl_) with high interfacial resistance (R_int_) of 869.4 Ω. These are attributed to adsorbing ions and transferred electrons in the electrolyte/electrode interface, demonstrating low charge propagation within the poorly formed double layer^21^. Finally, the vertical slope observed at lower frequencies is representative of a typical supercapacitor behaviour^22^.

The performance of the fibre-based supercapacitors was also accessed under bending through CV measurements at specific bending angles. Figure 4e shows the electrochemical behaviour of the device under several bending angles (0°, 30°, 60°, 90°, and 180°), while Fig. 4f shows an insignificant variation of the specific capacitance determined from the respective CV curves.

**Figure 5:**
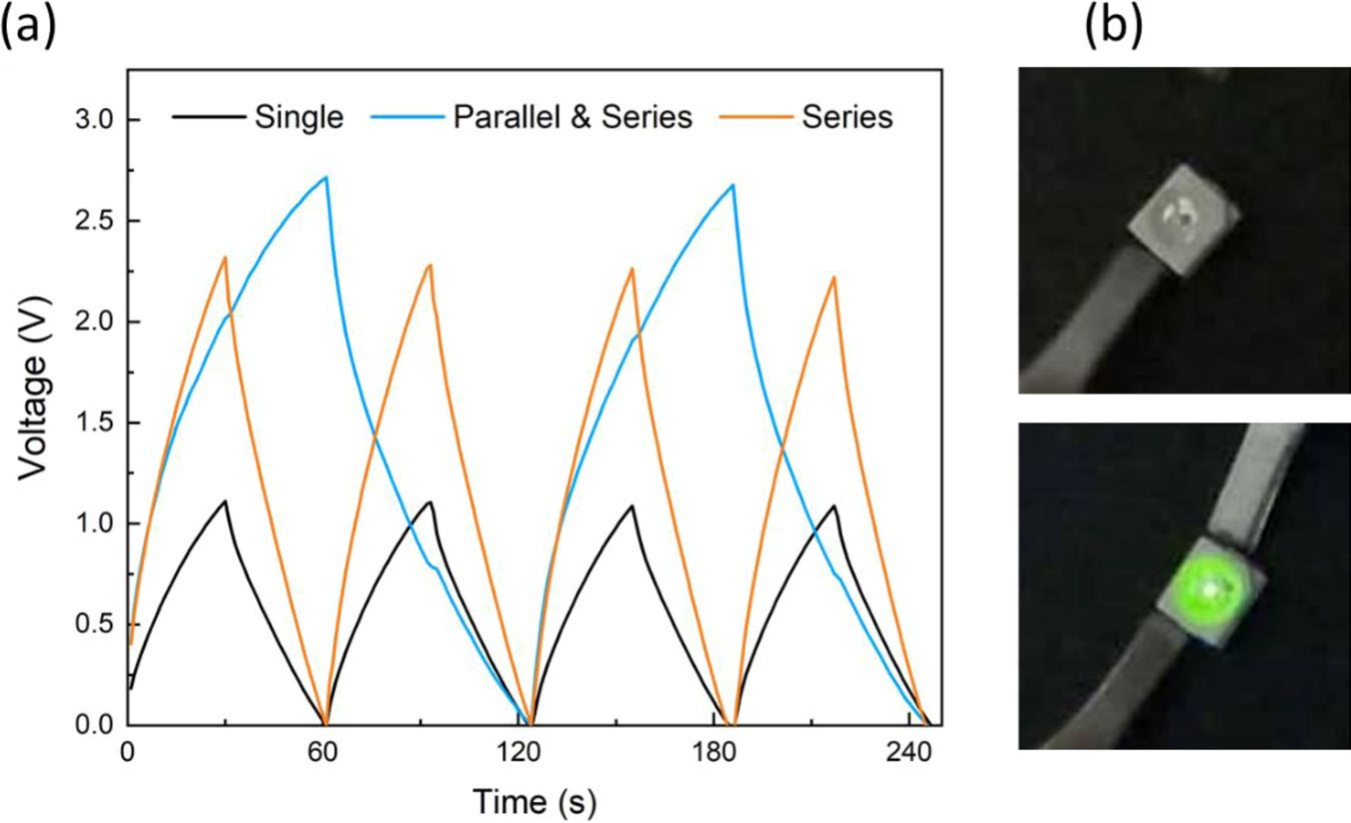
(**a**) Charge/Discharge cycles for several combination of supercapacitors: a single supercapacitor, two devices connected in series and four devices combined two in series with two in parallel; (**b**) The supercapacitors can turn on a green LED.

The results above demonstrate the high flexibility of the device without affecting its electrochemical performances which is a requirement for its use in wearable applications. The flexibility of the wire-based supercapacitor was also tested by repeatedly bending it from 0° up to 180° for 1000 cycles. After each set of 100 bending cycles, the CV performance was evaluated to determine the capacitance retention of CTf/CTf supercapacitor. As observed in Fig. 4g,h, the device preserves its original electrochemical performance after 1000 bending cycles which indicates its capability for withstanding repeated mechanical deformation, which is also a requirement for its application as a wearable device. When compared with other 1D supercapacitors found in the literature^23–27^, the electrochemical performances of the device here reported demonstrated comparable specific capacitance values and stability to mechanical deformation (Table 1). In order to demonstrate that the wire-based supercapacitors here reported are suitable for wearable applications, the devices were connected either in series or in a combination of series and parallel followed by charge/discharge measurements (Fig. 5a). A single device operates at 1.1 V while two supercapacitors connected in series exhibited a charge/discharge potential window of 2.3 V.

**Table 1:** Comparison of the electrochemical performances of several 1D supercapacitors found in literature with the one here reported.

Device	Areal Capacitance	Electrolyte	Geometry	Capacitance retention (bending cycles)	Ref.
PMMA wire/ZnO NWs/MnO_2_ and Kevlar fibre/ZnO NWs	2.4 mF.cm^−2^ (at 100 mV.s^−1^)	PVA/H_3_PO_4_	1D Twisted (Asymmetric configuration)	—	^23^
Stainless steel/Chinese ink and Active Carbon/Silver paint	3.18 mF.cm^−2^ (at 0.04 mA)	PVA/H_3_PO_4_/H_2_O	1D Core-Shell (Asymmetric configuration)	—	^24^
Aligned MWCNT/MnO_2_	3.16 mF.cm^−2^ (at 0.01 mA)	PVA/H_3_PO_4_	1D Twisted (Symmetric configuration)	—	^25^
Graphene fibres/PEDOT	15.39 mF.cm^−2^ (at 0.53 mA.cm^−2^)	PVA/H_2_SO_4_	1D Twisted (Symmetric configuration)	~100% after 300 cycles	^26^
CF/MnO_2_ and CF/MoO_3_	3.86 mF cm^−2^ (at 0.5 mA.cm^−2^)	PVA/KOH	1D Twisted (Asymmetric configuration)	89% after 3000 cycles	^27^
CT/Ppy	6.74 mF cm^−2^ (100 mV.s^−1^)	Simulated sweat solution	1D Twisted (Symmetric configuration)	~100% after 1000 cycles	**This work**

When four devices were connected by combining two in series with two in parallel, both output voltage and discharge time have double under the same charge/discharge current. Additionally, the devices have demonstrated to provide enough power to turn on a green light emitting diode (LED) that operates at a minimum voltage of 1.8 V. Four 4.5 cm-long supercapacitor wires connected two in series and two in parallel also enable to turn on a green LED for 8 s (Fig. 5b) - Supplementary information, Movie 1. These wire-based supercapacitors have demonstrated to be easily integrated to meet different power requirements and revealed to be promising to be weaved or knitted into electronic textiles.

## Conclusions

A novel symmetric fibre-shaped supercapacitor based on carbon threads functionalized with Ppy electrodes and a separator made of electrospun cellulose-based fibres was developed with the ability to use the wearer’s sweat as the electrolyte. In a twisted configuration, the number of twists done by the outer electrode around the separator was evaluated, since with their increase the electrochemical capacitance of devices also increases. A stable cycling performance was observed by submitting the device to over 1300 consecutive cycles to CD measurements at 78 mA.g^−1^ displaying capacitance retention of almost 100%. The CTf/CTf structure showed a specific capacitance of 2.3 F.g^−1^, an energy of 386.5 mWh.kg^−1^ and a power density of 46.4 kW.kg^−1^. The device also showed excellent flexibility with no evident degradation of electrochemical performances after 1000 bending cycles at 180°.

Finally, four wire-based supercapacitors were combined in series and in parallel demonstrating their ability to turn on a green LED. Due to their electrochemical and mechanical stability, the yarn-based supercapacitor here reported can be a promising storage device for skin-like wearable electronics applications.

## Supplementary information

Supplementary movie 1

Supplementary information.
