# Supplementary material for: Carbon threads sweat-based supercapacitors for electronic textiles
Source: Sci Rep. 2020 May 7;10:7703. doi: 10.1038/s41598-020-64649-2 (PMC7206100; doi:10.1038/s41598-020-64649-2)
Supplement: Supplementary file 2 — Supplementary information. [file 41598_2020_64649_MOESM2_ESM.docx]

Supporting information

**Carbon threads sweat-based supercapacitors for electronic textiles**

Nuno Lima, Ana C. Baptista*, Bruno M. Morais Faustino, Sofia Taborda, Ana C. Marques and Isabel Ferreira

*Corresponding Author: anacbaptista@fct.unl.pt (Ana C. Baptista)

**Designing of the electrospinning target for wire support**


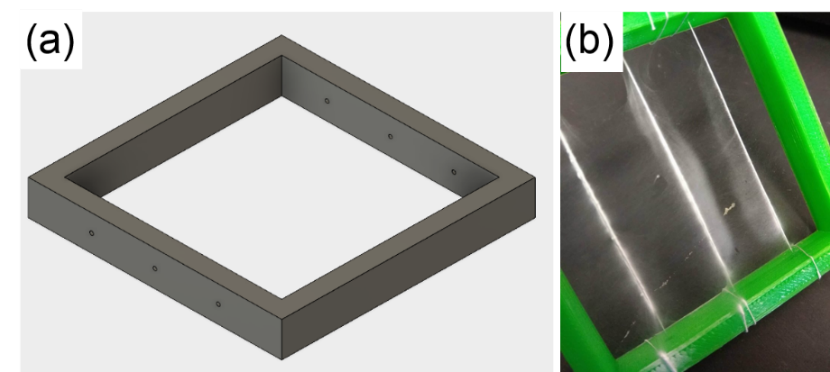


**Figure SI 1** – (a) Schematic of the frame design for 3D printing the wire support for electrospinning setup and (b) image of the printed frame with several wires coated with electrospun cellulose acetate fibres.

**Assembly of the supercapacitors with twisted configuration**

**
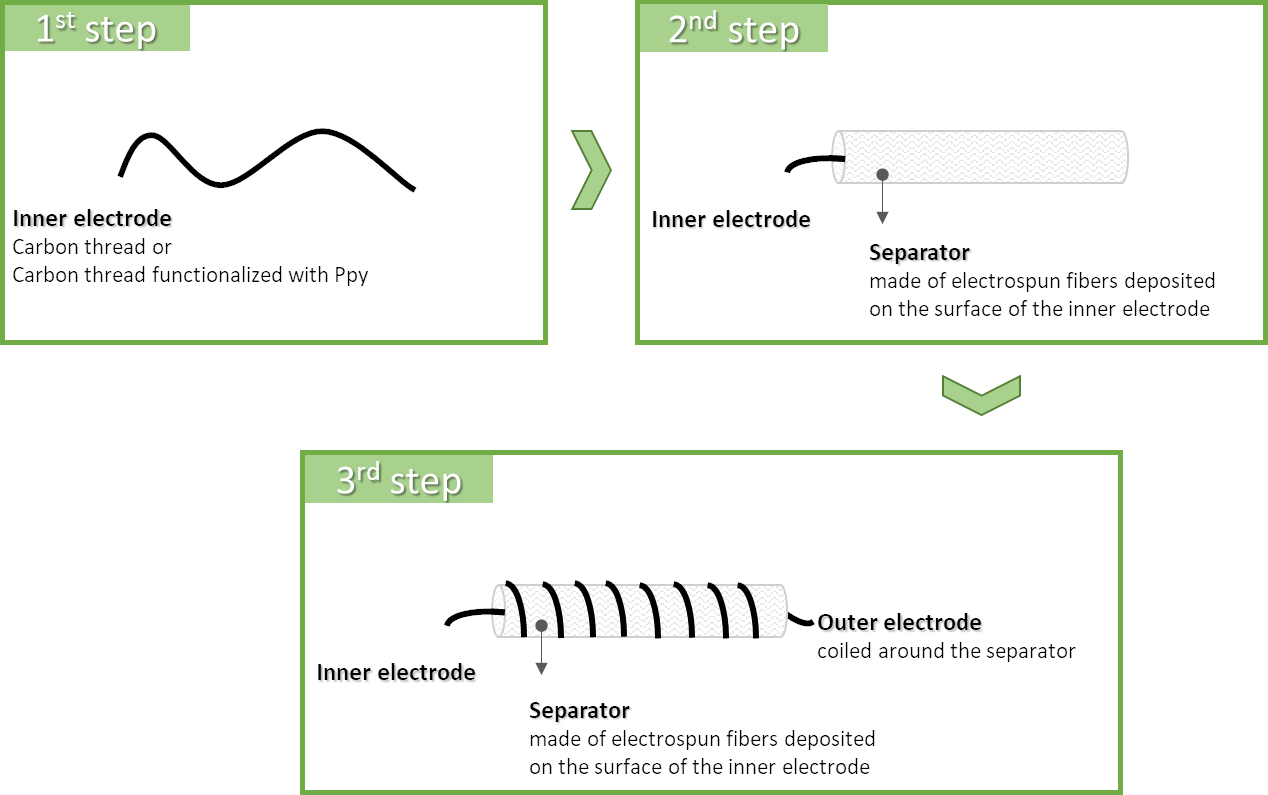
**

**Figure SI 2 –** A detailed schematics about the wire-based supercapacitor assembly: The inner electrode is a commercial conductive carbon-thread that depending on the configuration was or not functionalized with Ppy or not (1^st^ step); then, cellulose acetate electrospun fibres were deposited on the surface of the carbon thread forming the separator (2^nd^ step); finally, other conductive carbon thread is twisted around the separator forming the outer electrode (3^rd^ step). Several combinations of inner/outer electrodes were studied: carbon thread/carbon thread (labelled by CT/CT); carbon thread functionalized with Ppy/carbon thread functionalized with Ppy (labelled by CTf/CTf), and an asymmetrical configuration of carbon thread functionalized with Ppy/carbon thread (labelled by CTf/CT).

**Setup used for electrochemical measurements**


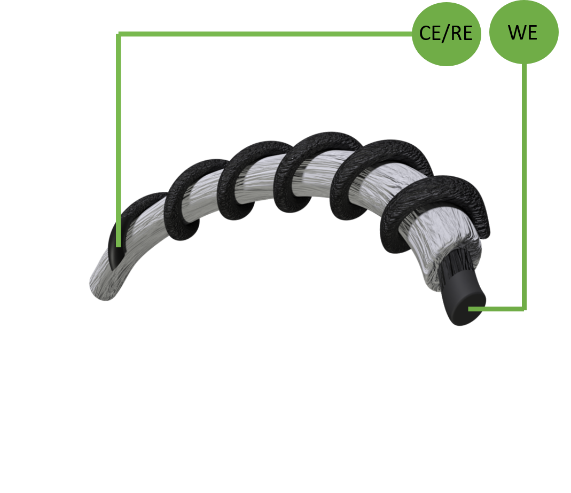


**Figure SI3** – Schematic of the the two-electrode electrochemical characterization setup. Where CE is the counter electrode, RE is the reference electrode and WE is the working electrode.

**Detailed morphological analysis of carbon thread coated with Ppy:**

**
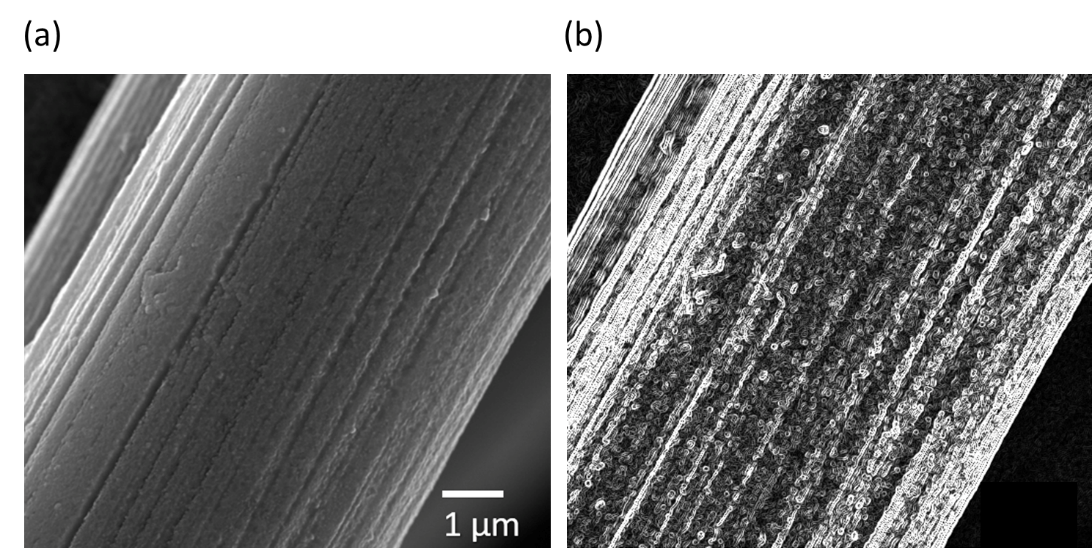
**

**Figure SI4** – SEM image of carbon thread uniformly coated with Ppy: (a) original image and (b) after imaging processing with IMAGE J software using a filter for edge detection.

**Electrolyte study evaluation:** Simulated Sweat Solution and KOH

**Table SI1.** Specific capacitance obtained at 100 mV.s^-1^ for CT/CT, CTf/C and CTf/CTf supercapacitors using simulated sweat solution (SSS) and KOH as electrolytes. Each supercapacitor was impregnated with 40 μl of electrolyte and the outer electrode was twisted three times around the separator (twisting number = 3).

|  | **KOH** | **SSS** |
| --- | --- | --- |
| **CT/CT** | 38 mF.g ^-1^ | 46 mF.g ^-1^ |
| **CTf/CT** | 105 mF.g ^-1^ | 100 mF.g ^-1^ |
| **CTf/CTf** | 1609 mF.g ^-1^ | 2353 mF.g ^-1^ |


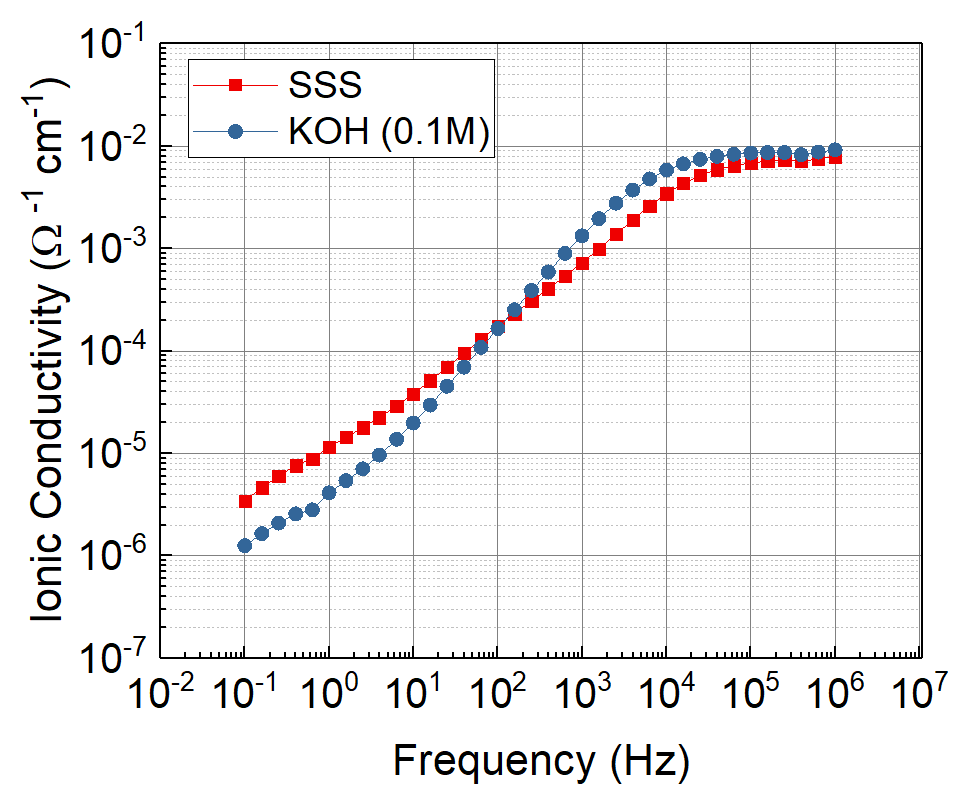


**Figure SI5** – Bode plot showing ionic conductivity of the SSS (red square) and KOH (0.1M) (blue dot) electrolytes in a 100 µm thick CA membrane, for different frequencies. Both were tested with the same setup.

**LED experiment movie**

Please see Movie 1
